# Pulchinenosides from Pulsatilla Chinensis Increase P-Glycoprotein Activity and Induce P-Glycoprotein Expression

**DOI:** 10.1155/2020/4861719

**Published:** 2020-02-18

**Authors:** Yali Liu, Ling Zhang, Shaofeng Wei, Jinyang Cai, Zhenzhong Zang, Meng Wang, Dan Su, Phillip M. Gerk

**Affiliations:** ^1^Jiangxi University of Traditional Chinese Medicine, 818 Meiling Road, Nanchang 330006, China; ^2^Science and Technology College, Jiangxi University of Traditional Chinese Medicine, 818 Meiling Road, Nanchang 330006, China; ^3^Virginia Commonwealth University, MCV Campus, 410 N. 12th Street, Richmond, VA 23298–0533, USA

## Abstract

Five pulchinenosides (pulchinenoside B3, pulchinenoside BD, pulchinenoside B7, pulchinenoside B10, and pulchinenoside B11) isolated from Pulsatilla chinensis (Bge) Regel saponins extract exhibited strong antitumor activities but poor gastrointestinal absorption properties. The enteric induction of P-glycoprotein (P-gp) is understood to restrict the oral bioavailability of some pharmaceutical compounds and lead to adverse drug reactions. Therefore, the present investigation was intended to delineate the impacts of pulchinenosides on cellular P-gp function and expression using Sf9 membrane vesicles and LS180 cells as a surrogate of human intestinal epithelial cells. Preliminary cytotoxic studies showed that 10 *μ*M was an acceptable concentration for cytotoxicity and antiproliferation studies for all pulchinenosides using the alamarBlue assay. The cell cycle of LS180 cells detected by flow cytometry was not significantly influenced after 48 hours of coincubation with 10 *μ*M of pulchinenosides. In the presence of pulchinenosides, the ATP-dependent transport of N-methyl-quinidine mediated by P-glycoprotein was stimulated significantly. The upregulation of P-glycoprotein and mRNA levels was found by Western blot and real-time PCR analysis in LS180 cells. Parallel changes indicate that all pulchinenosides are exposed to pulchinenosides-mediated transcriptional regulation. In conclusion, pulchinenosides could induce P-glycoprotein expression and directly increase its functional activity.

## 1. Introduction

A febrifugal traditional Chinese medicine named Pulsatilla chinensis (Bge) Regel has been used in medicinal and traditional practices in China and other Eastern countries for centuries. There are 13 Chinese patent medicine formulas and 49 ancient prescriptions containing Pulsatilla chinensis retrieved on the Database of Drug Intelligence Network-Traditional Chinese medicine prescription [1]. It has been already recorded in the Chinese Pharmacopeia for ages and portrayed as “heat-clearing and detoxification, blood-cooling, and diarrhea-preventing [2].” P. chinensis has been extensively utilized to treat amoebic ailments, vaginal trichomoniasis, microbial diseases, and bacillosis [3]. Now, more and more attention is being paid to its antitumor activities [4–7].

As previously reported, Pulsatilla chinensis saponins (PRS) extract was considered to possess biologically active components that exert potential antitumor activity in nude mice without producing toxicity [8]. Some constituents of Pulsatilla were identified and isolated in ceaseless efforts to find potential biologically active constituents from the Pulsatilla chinensis saponin extract. It was determined that five oleracea pulchinenosides (pulchinenoside B3, pulchinenoside BD, pulchinenoside B7, pulchinenoside B10, and pulchinenoside B11) were major saponins present in the saponin extract [7]. In contrast to anemoside B4, which was described in Chinese Pharmacopeia [2], recent reports revealed that these five pulchinenosides probably explain the antitumor activity of PRS more effectively [9].

**Figure 1:**
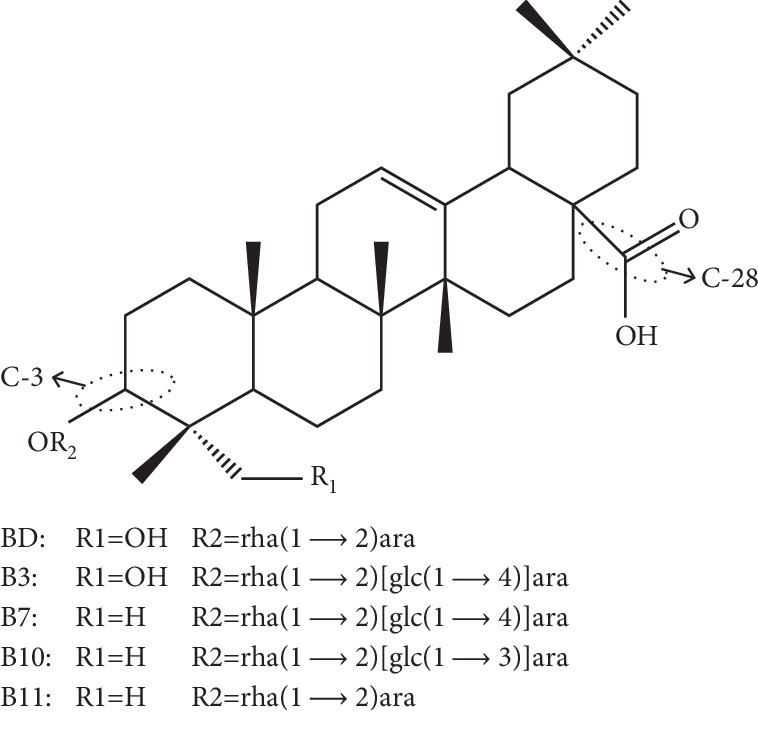
Chemical structures of pulchinenosides.

These five pulchinenosides are pentacyclic triterpenoids (Figure 1). They are all classified as monodesmosidic depending on the quantity of sugar chain presence [10]. They are sorted into hederagenin saponins (pulchinenosides B3 and BD) and oleanolic acid saponins (pulchinenosides B7, B10, and B11) depending on types of sapogenin. The structure-activity relationship of two oleanolic saponins has been explored, and it has been shown that the anticancer activity is related to the 28-carboxylic acid and the glycosidic linkage on C-3 [11]. In this study, all five pulchinenosides (pulchinenoside B3, pulchinenoside BD, pulchinenoside B7, pulchinenoside B10, and pulchinenoside B11) researched include these dynamic structural characteristics.

Interestingly, our previous research found that the bioavailability of these pulchinenosides B3, BD, B7, B10, and B11 in rats was only 1.16%, 1.17%, 0.55%, 0.96%, and 2.50%, respectively, thus exhibiting poor gastrointestinal absorption [12]. Despite extremely low bioavailability, they inhibited the liver tumour growth up to 60% *ex vivo* and *in vivo* [7]. These results suggested that the gastrointestinal absorption barrier, which is probably the main factor limiting its maximum pharmacodynamic effect, cannot be ignored.

A transmembrane protein called P-glycoprotein is a part of the ATP-binding cassette (ABC) transporter superfamily and pumps various chemical substances, including drugs, xenobiotics, natural substances, insecticides, and peptides, out of many cell types using the energy from hydrolysis of ATP [13, 14]. P-glycoprotein contributes to low and variable oral bioavailability or tissue distribution of its substrates [15]. P-glycoprotein is extensively expressed in the apical membranes of several epithelial tissues necessary for xenobiotic disposition, including intestine, liver, and kidney, as well as the blood-brain barrier [16, 17].

The P-glycoprotein expression or function may be induced by some compounds, which then affect the absorption and bioavailability of P-gp substrates themselves. Examples include Chinese patent medicine Siwu decoction [18], *Radix Glycyrrhizae* and its main components [19], and many other bioactive natural products [13]. In the light of herb-drug interaction, the herbal treatment Saint *John's* wort which induces both P-gp and CYP3A4 expression, resulting in a decrease in the concentration of cyclosporin in blood and transport rejection in some cases [20, 21].

Recently, our research has identified pulchinenosides as substrates of P-glycoprotein in rat intestinal segments [22]. Since this suggested a crucial relationship in low drug bioavailability and P-glycoprotein function, herein, we explore the impacts of pulchinenosides on P-glycoprotein function, which could restrict their absorption. Also, since certain P-glycoprotein function regulators may affect its expression, it is vital to determine the impact of pulchinenosides on P-glycoprotein function and their effect on P-glycoprotein expression. The purpose of this research was to describe the impact of pulchinenosides on the functional activity and expression of P-glycoprotein in LS180 cells and P-glycoprotein overexpressing *Sf*9 insect cell membrane vesicles as a substitute for human intestinal epithelial cells.

## 2. Materials and Methods

### 2.1. Materials

The passage 38 LS180 cells were purchased from ATCC (Manassas, VA, USA). The materials such as Dulbecco's minimal essential medium (DMEM), fetal bovine serum (FBS), nonessential amino acids (NEAA), and optimum minimal culture medium were from Gibco BRL Life Technologies (Grand Island, New York, USA). Cell culture vessels were from NUNC (Rochester, New York, USA). SuperSignal West Pico enhanced chemiluminescence was purchased from Pierce Incorporation (Rockford, Illinois, USA). Sodium lauryl sulfate and 2-mercaptoethanol were from Merck (Darmstadt, Germany). *Sf*9 membrane vesicles containing or lacking recombinant human P-gp were from Corning Incorporation (Corning, NY, USA). Polyvinylidene fluoride membranes were from Millipore Corporation (Billerica, Massachusetts, USA). Protein determination dye reagent, tetrabromophenol blue, dithiothreitol, acrylamide/dual solution, and polyethylene glycol octyl phenyl ether were all from Bio-Rad Laboratories Incorporation (Hercules, CA, USA). The antibody used for MDR-1 (sc-13131) and tubulin (sc-398103) was purchased from Santa Cruz Biotechnology Incorporation (Dallas, Texas, USA). Dimethyl sulfoxide, streptomycin sulfate, Hank's balanced salt solution, HEPES, albumin bovine, digoxin, Kodak film, resorufin, and EDTA were all purchased from Sigma-Aldrich Corporation (Saint Louis, Missouri, USA). Resazurin (which gets metabolized to resorufin in the alamarBlue assay) was purchased from Acros Organics (Fair Lawn, NJ, USA). The RNeasy mini kit for extracting RNA was from Bioline (London, UK).

The presented standard five pulchinenosides (pulchinenoside B3, pulchinenoside BD, pulchinenoside B7, pulchinenoside B10, and pulchinenoside B11) and hederagenin (purity >95%) were separated in the Department of Natural Medicine (NPEC, Nanchang, People's Republic of China). They were identified by spectral analytical means, containing the optical spectroscopy of MS, ^1^H-NMR, and ^13^C-NMR. The information conforms to those documented in the literature [23, 24].

2.5 kg of the desiccated plant material was extracted three times with 70% EtOH and 30% of water beneath the reflux condition. The extractives were then dried under reduced pressure. 280 g of residue was utilized for performing chromatography using a D101 cationic column, and the elution used a water-EtOH gradient. Fractions were eluted with 60% EtOH, combined, freeze-dried, and it gained 125 g of PRS powder [12]. These components (BD, B3, B7, B10, and B11) in PRS extracts were analyzed on HPLC and found to be 24.1%, 7.4%, 12.4%, 13.5%, and 8.5%, respectively, representing 65.9% of absolute saponins.

### 2.2. Cell Culture

The passage 52–60 of LS180 cells were cultivated in 15 mL of DMEM (containing 10% fetal bovine serum and 1% nonessential amino acids) in 75 cm^2^ of cell culture vessels. These cells were grown at 37°C with 5% carbon dioxide and 95% atmosphere in a carbon dioxide incubator, and the medium was changed every second day until these cells achieve 80–90% confluence, generally in three to four days. Cells were passaged in the absence of trypsin by scraping then passing 6 cycles through a 23G × 1 needle.

### 2.3. Cytotoxicity and Antiproliferation

The alamarBlue assay quantifies the mitochondrial activity of living cells through the reduction of resazurin to resorufin [25]. This assay was applied to determine the cytotoxic and antiproliferative effects of pulchinenosides on LS180 cells. For cytotoxicity research, in brief, LS180 cells were cultivated in the 96-well plates for 48 h (seeding density: 10^4^ cells/well). Solutions of pulchinenosides in DMSO were diluted with DMEM to get concentrations of 0–100 *μ*M pulchinenosides and maintained a constant 0.5% DMSO. Control specimens contained 0.5% of DMSO in DMEM and 0.1% of sodium lauryl sulfate in DMEM. The experiment was started by substituting the culture medium in each well with 100 *μ*L of control solution or treatment medium and incubating the cells for 4 h at 37°C in a 5% carbon dioxide incubator. Resazurin (220 *μ*M) was added by a Synergy 2 microplate reader (Bio-Tek; Winooski, VT) in an amount equivalent to 10% of the cultivated volume, and the plate was shaken and warmed for 2 min to start the reaction. Resorufin fluorescence was measured over 10 min at excitation 530 nm and emission 590 nm, and slopes were determined using Gen5 software (Bio-Tek). Cellular viability was calculated as the ratio of the slopes for the cells treated with pulchinenosides versus control (no pulchinenosides).

For antiproliferative effects, LS180 cells were plated and allowed to attach on the 96-well plates for 48 h. Next, the medium in every well was replaced with media containing 0–100 *μ*M pulchinenosides. The cell plate was incubated for an additional 24 h, and the alamarBlue assay was performed as described above.

### 2.4. Cell Cycle

LS180 cells (5 × 10^5^ cells/well) were plated on 6-well plates and cultivated for 24 h in the carbon dioxide incubator with 2 mL of DMEM medium. This medium was replaced with medium containing 10 *μ*M of pulchinenosides and incubated for an additional 24 to 48 h. Attached cells were trypsinized, washed with cold phosphate buffer saline, followed by 75% EtOH, and stored at −20°C. Cells were rinsed again with phosphate-buffered saline before staining (ribonuclease A: 200 mg/mL, propidium iodide (PI), Triton X-100 in phosphate buffer saline: 0.1%). The analysis of cells on the flow cytometry instrument (Guava Technologies Inc., California, USA) was performed via the FITC channel, and 5 × 10^3^ cells were collected each time.

### 2.5. Transport Study

The *Sf*9 membrane vesicle assay has been adopted for a transport study [26, 27]. In brief, *Sf*9 insect cell membrane vesicles lacking (control) or overexpressing human P-glycoprotein were obtained from Optivia Biotechnology (Santa Clara, CA). The complete buffer contained 5 mM adenosine triphosphate (ATP) or adenosine monophosphate (AMP), creatine phosphokinase (100 *μ*g/mL), creatine phosphate (10 mM), Tris HCl (10 mM) at pH 7.4, sucrose (250 mM), MgCl_2_ (10 mM), N-methyl-quinidine (final concentration 10 *μ*M), and 10 *μ*M pulchinenosides in DMSO (concentration up to 0.5% v/v). The prewarmed solutions were mixed with P-glycoprotein or control membrane vesicles (final concentration 0.5 *μ*g/mL) and incubated at 37°C for 5 min and then transferred to a Millipore Multiscreen filter plate (Millipore: MAHVN4510, Milford, MA). Each well was aspirated and then rinsed three times with iced buffer, including 10 mM Tris HCL in pH 7.4, 250 mM sucrose, and 100 mM sodium chloride. N-methyl-quinidine was eluted from the filters with 92% ACN and 8% water.

Quantification of N-methyl-quinidine [28] was performed on a Waters 2695 HPLC system (Waters Technologies Inc., Massachusetts, USA) with a Waters 2475 fluorescence detector (excitation wavelength 330 nm and excitation wavelength 400 nm), using Phenomenex C_18_ column (4.6 × 150 mm, 3 *μ*m). The HPLC method comprised an isocratic mobile phase flow (1 mL/min) of 75% methanol and 25% aqueous (0.5% formic acid and 0.1% triethylamine). Nonspecific binding of N-methyl-quinidine was corrected by subtracting the values in the absence of ATP (substituted with AMP), and *Sf*9 endogenous transport activity was corrected by subtracting the control membrane values, thus yielding values for ATP-dependent and P-glycoprotein-mediated transport activity.

The assay for pulchinenosides in rat plasma was validated following the guidance for the bioanalytical methods validation [29].

### 2.6. Western Blot Analysis of P-gp

After treatment, the LS180 cells were harvested, and whole-cell pellets were lysed by sonication in 1x sample buffer (200 mM Tris-HCl at pH 6.8, 2% sodium dodecyl sulfate, 10% glycerol, 0.2% bromphenol blue, and proteinase inhibitor cocktail) and boiled for 10 min. Equivalent amounts of protein lysates were placed in 8% SDS PAGE gels and separated; then, the protein products were electrophoretically transferred to PVDF films (Millipore, Billerica, MA). After incubation for 60 min with 5% skims milk in phosphate-buffered saline with Tween-20 (PBST), the film was rinsed once with PBST, and the primary antibody was gently rocked overnight at 4°C. Membranes were then rinsed 3 times for 5 min and hatched with anti-rabbit or HRP-conjugated anti-mouse antibody diluted 1 : 5000 at room temperature for 1 h. The blot was rinsed in PBST 3 times and developed by an ECL system (Pierce, ThermoFisher). The blot was performed 3 times, and densitometry was quantitated using NIH ImageJ software.

### 2.7. Real-Time RT-PCR Analysis of ABCB1 mRNA

Total RNA was extracted from LS180 cells using the ISOLATE RNA mini kit (Bioline, cat. no. BIO-52072). 1 *μ*g of total RNA was applied to cDNA reverse transcription using a kit (Applied Biosystems, cat. no. 4368814). The SYBR Green main mixtures (Applied Biosystems, cat. no. A25742) were applied to real-time PCR, and the results were analyzed using QUANT-STUDIO 3.0 software. ABCB1 mRNA abundance was standardized to the expression of glyceraldehyde-phosphate dehydrogenase (GAPDH). The primers applied to PCR are as follows:

  5′-CTGCTTGATGGCAAAGAAATAAAG-3′ (forward) and
  5′-GGCTGTTGTCTCCATAGGCAAT-3′ (reverse) for MDR-1 and
  5′-AATCCCATCACCATCTTCCA-3′ (forward) and
  5′-TGGACTCCACGACGTACTCA-3′ (reverse) for GAPDH.

### 2.8. Statistical Analyses

Data were plotted as average value ± SD applying GraphPad Prism 7.00 (GraphPad Software Incorporation, La Jolla, California, USA). One way ANOVA (Dunnett's) postanalysis and two way ANOVA (Bonferroni's) postanalysis were used for comparisons (^*∗*^*P* < 0.05). Nonlinear regression (Prism) was used to determine IC_50_ values.

## 3. Results

### 3.1. Cytotoxicity and Antiproliferative Activity

**Figure 2:**
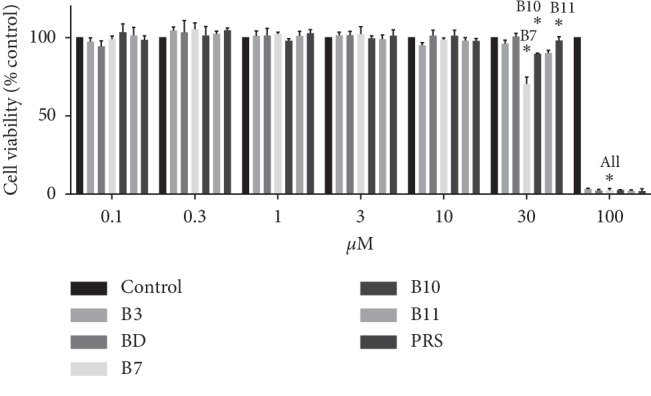
Effect of different concentrations of pulchinenosides on the viability of LS180 cells as assessed by alamarBlue assay after 4 hours of incubation (*n* = 3, mean ± SD). ^*∗*^*P* < 0.05.

**Figure 3:**
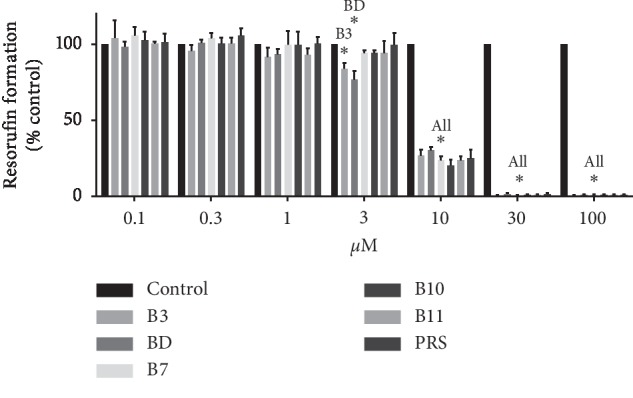
Effects of different concentrations of pulchinenosides on the proliferation of LS180 cells as assessed by alamarBlue assay after 72 hours of incubation (*n* = 3, mean ± SD). ^*∗*^*P* < 0.05.

After 4 h exposure to pulchinenosides (up to 100 *μ*M), significant toxicity was observed at concentrations of 100 *μ*M for B3, BD, and PRS and 30 *μ*M for B7, B10, and B11 (Figure 2). The proliferation of LS180 cells was also dependent upon pulchinenosides concentrations. Although cell proliferation was not affected by pulchinenosides at 1 *μ*M for B3 and BD and 3 *μ*M for B7, B10, B11, and PRS, cell viability was affected significantly at 3 *μ*M for B3 and BD and at 10 *μ*M for B7, B10, B11, and PRS, respectively. IC_50_ values for B3, BD, B7, B10, B1, and PRS were 4.13 ± 0.45, 7.05 ± 0.52, 5.77 ± 0.36, 7.49 ± 0.46, 8.78 ± 0.68, 9.78 ± 0.27 *μ*M, respectively. Therefore, for all these compounds, 10 *μ*M was chosen to evaluate the effects of the compounds on the cell cycle (Figure 3).

### 3.2. Cell Cycle

**Figure 4:**
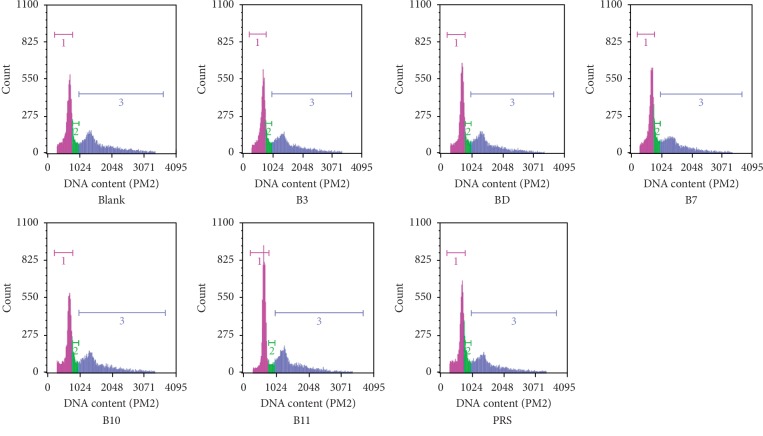
DNA content of cell cycle distributed on LS180 cells cocultivated with pulchinenosides (10 *μ*M) for 48 hours. Blank is the negative control; B3, BD, B7, B10, B11, and PRS represent pulchinenosides as described.

**Figure 5:**
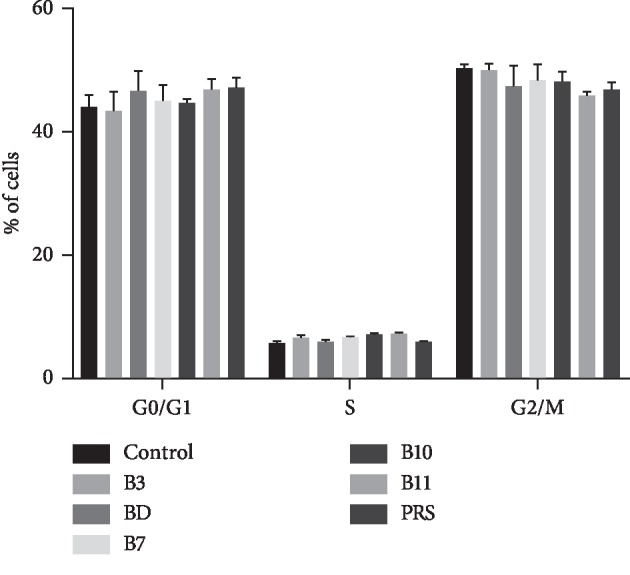
Cell cycle distributed on LS180 cells cultivated with pulchinenosides for 48 hours (*n* = 3, mean ± SD). ^*∗*^*P* < 0.05.

The cell cycle progression, however, was relevant with a substantial change in proliferation rate, as shown in (Figures 4 and 5). After 48 h of exposure to 10 *μ*M pulchinenosides, there was no significant effect on cycle distribution.

### 3.3. Transport Study

**Figure 6:**
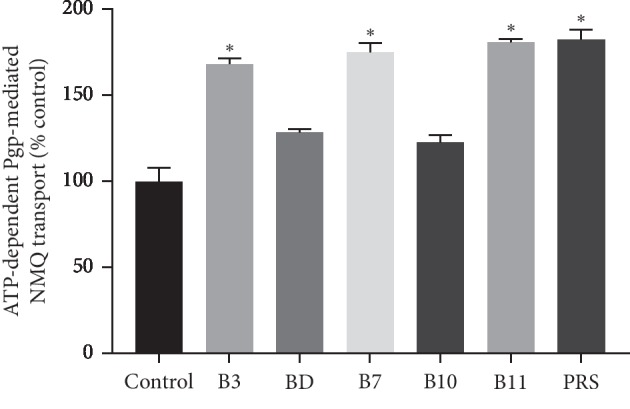
The effects of pulchinenosides (10 *μ*M) on P-glycoprotein transport activity (*n* = 3, mean ± SD). ^*∗*^*P* < 0.05.

Transport studies showed that the ATP-dependent, P-glycoprotein-mediated accumulation of N-methyl-quinidine (NMQ) in the inside-out membrane vesicles increased significantly in the presence of 10 *μ*M concentrations of B3, B7, B11 and PRS as shown in (Figure 6). These results indicate that 10 *μ*M stimulated the transport of N-methyl-quinidine by P-glycoprotein pulchinenosides nearly two-fold for B11 and PRS.

### 3.4. P-Glycoprotein Expression

**Figure 7:**
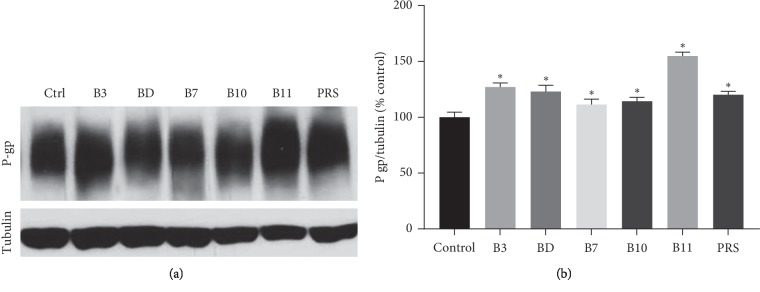
P-glycoprotein expression levels were cultivated in 6-well plates and exposed to 10 *μ*M pulchinenosides for 48 hours on LS180 cells. (a) Representative western blots of P-gp and tubulin. (b) Densitometry scans of western blots (*n* = 3, mean ± SD). ^*∗*^*P* < 0.05.

Western Blot determined P-glycoprotein expression in the LS180 cells plated on the 6-well plate. On day 9, cells were exposed to 10 *μ*M of various pulchinenosides for 48 h. As indicated in (Figure 7), all pulchinenosides significantly upregulated the membrane expression level of P-glycoprotein in cells.

### 3.5. ABCB1 mRNA Expression

**Figure 8:**
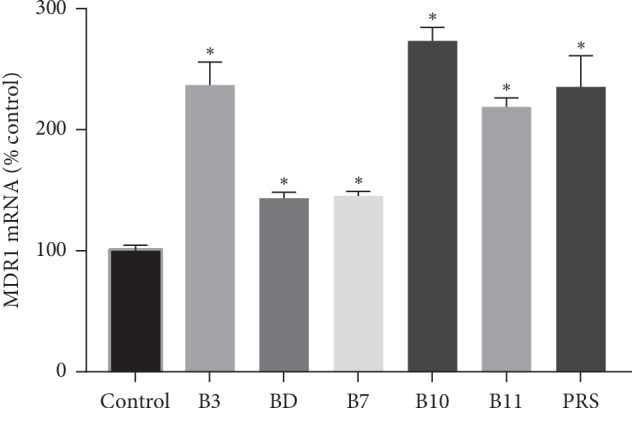
RT-polymerase chain reaction analysis of multidrug resistance gene 1 mRNA in LS180 cells exposed to pulchinenosides for 48 hours (*n* = 3, mean ± SD). ^*∗*^*P* < 0.05.

ABCB1 mRNA in the LS180 cells cultivated on the 6-well plate was quantified, applying real-time RT-PCR analysis standardized to the blank control. It suggested that all the pulchinenosides, regardless of the type of saponin, upregulated the ABCB1 mRNA expression on LS180 cells (Figure 8), and all pulchinenosides have significant differences, especially for B3, B10, B11, and PRS, which induced ABCB1 mRNA transcription more than 2-fold that of control. These results suggested a pulchinenosides-mediated transcriptional modulation.

## 4. Discussion

Intestinal P-gp plays a crucial role in the absorption and excretion of oral xenobiotics and drugs. The changes in P-glycoprotein/ABCB1 activity due to its induction and/or inhibition will lead to drug-drug or herbal-drug interaction that can change pharmacodynamics and drug response [30, 31]. Thus, compounds which affect the functional activity and expression of P-gp are vital from the pharmaceutical and toxicological perspective [32]. Within this research, we assessed the effects of pulchinenosides exposure on P-glycoprotein function in *Sf*9 membrane vesicles and expression in LS180 cells. To our knowledge, this is the first report to examine the influence of pulchinenosides, an anticancer traditional Chinese herbal drug, on both the P-gp transport activity and expression in LS180 human intestinal cells.

In bioavailability research, a chemical compound is usually administered orally or intravenously, which is the primary method for deciding the definite bioavailability value of a single chemical drug [26]. While keeping in mind the multifaceted nature of TCMs [23], different components may interact. Thus, when estimating their bioavailability or studying the mechanisms involved, it is better to study the compounds in combination(s) as well as individually. That is why, we studied not only purified compounds (B3, BD, B7, B10, and B11) but also mixed compounds (PRS).

The cytotoxic effects of pulchinenosides were also examined in this project. At 30 *μ*M concentrations of B3, BD, and PRS and at 10 *μ*M of B7, B10, and B11, pulchinenosides did not show cytotoxic effect after 4 h exposure. Pulchinenosides B3 and BD (3 *μ*M) and B7, B10, B11, and PRS (10 *μ*M) reduced cell proliferation when the exposure cycle was extended to 72 h. Compared with the control group, pulchinenosides did not have a significant influence on cell cycle progression.

The P-gp expression is generally influenced by many *in vitro* and *in vivo* factors, which include extracellular matrix, growth factors, arsenite, heat shock, partial hepatectomy, protein kinase C agonists, sodium butyrate, and even some of its inhibitors and substrates [33]. In our study, pulchinenosides apparently stimulated the ABCB1/P-gp transport of N-methyl-quinidine. This result suggests that pulchinenosides may have the potential to reduce the intestinal absorption of P-glycoprotein substrates. There are many drugs that are usually considered P-gp substrates, but they can also upregulate the expression of ABCB1/P-gp [34, 35]. We had previously demonstrated that pulchinenosides were substrates of P-glycoprotein, and their movement across the rat intestinal epithelium was directed by P-glycoprotein-mediated efflux activities [22]. In the present study, we have shown that pulchinenosides (especially, B3, B7, B11, and PRS) at 10 *μ*M concentrations were capable of significantly stimulating P-glycoprotein transport activity. As a result, the enhanced expression and activity of P-glycoprotein could inhibit the absorption of such components and limit their bioavailability [36].

Cells can respond to the existence of xenobiotics via upregulating drug efflux pumps, such as P-gp, to prolong their survival. In terms of the influence of pulchinenosides on P-glycoprotein function, 48 h exposure to pulchinenosides appeared to consistently increase P-glycoprotein expression in the LS180 cells, although the mechanism has yet to be identified. In contrast to most steroidal saponins [37] that might downregulate P-gp expression, pulchinenosides exposure leads to the upregulation of P-gp/ABCB1 state. Potential mechanisms involved within the induction of P-gp functional activity, and expression could involve the Pregnane x Receptor (PXR) [38], protein kinase C [39], and the initiation of the NF-kB [40] and the PI3-kinase/Akt [41] pathways. PXR activation is among the potential pathways associated with the transcriptional initiation of the ABCB1 expression. Certain compounds such as anticancer drugs, cholesterol-lowering statins, plant extracts, rifampin, and HIV protease inhibitors are among the PXR ligands [42–44]. The dietary phytochemicals tangeretin and gingkolides A and B significantly induced expression and activity of P-gp in LS180 cells through PXR [45]. Furthermore, P-gp is also induced by 1*α*,25-dihydroxy vitamin D3 through the vitamin-D receptor, meanwhile, also inducing cytochrome P450 3A4 [46]. Our data showing induction of P-gp expression by pulchinenosides are consistent with these previous studies, although further experiments are needed to determine the regulatory mechanism.

## 5. Conclusion

In summary, this research has indicated that to differing extents, pulchinenosides can stimulate P-glycoprotein functional activities and upregulate P-glycoprotein/ABCB1 mRNA and protein expression. Further research is required to clarify the mechanism of increased P-glycoprotein activity and expression via regulatory pathways. These results suggest that both short-term and long-term dosing of pulchinenosides has the potential to reduce the oral bioavailability of P-glycoprotein substrates.

## Data Availability

The data used to support the findings of this study are available from the corresponding author upon request.

## Abbreviations

P-gp: P-glycoprotein
*Sf*9: *Spodoptera frugiperda*
HPLC: High-performance liquid chromatography
ATP: Adenosine triphosphate
RT-PCR: Reverse transcriptase-polymerase chain reaction
ABCB1: Multidrug resistance protein 1.
